# Metastasis-Induced Acute Pancreatitis Successfully Treated with Chemotherapy and Radiotherapy in a Patient with Small Cell Lung Cancer

**DOI:** 10.1155/2015/304279

**Published:** 2015-05-14

**Authors:** Kerem Okutur, Mustafa Bozkurt, Taner Korkmaz, Ercan Karaaslan, Levent Guner, Suha Goksel, Gokhan Demir

**Affiliations:** ^1^Department of Medical Oncology, Acibadem University School of Medicine, Buyukdere Caddesi, No. 40, Sariyer, 34453 Istanbul, Turkey; ^2^Department of Medical Oncology, Acibadem Maslak Hospital, Buyukdere Caddesi, No. 40, Sariyer, 34453 Istanbul, Turkey; ^3^Department of Radiology, Acibadem University School of Medicine, Buyukdere Caddesi, No. 40, Sariyer, 34453 Istanbul, Turkey; ^4^Department of Nuclear Medicine, Acibadem University School of Medicine, Buyukdere Caddesi, No. 40, Sariyer, 34453 Istanbul, Turkey; ^5^Department of Pathology, Acibadem Maslak Hospital, Buyukdere Caddesi, No. 40, Sariyer, 34453 Istanbul, Turkey

## Abstract

Although involvement of pancreas is a common finding in small cell lung cancer (SCLC), metastasis-induced acute pancreatitis (MIAP) is very rare. A 50-year-old female with SCLC who had limited disease and achieved full response after treatment presented with acute pancreatitis during her follow-up. The radiologic studies revealed a small area causing obliteration of the pancreatic duct without mass in the pancreatic neck, and endoscopic ultrasound-guided fine-needle aspiration (EUS-FNA) confirmed the metastasis of SCLC. The patient was treated successfully with systemic chemotherapy and radiotherapy delivered to pancreatic field. In SCLC, cases of MIAP can be encountered with conventional computed tomography with no mass image, and positron emission tomography and EUS-FNA can be useful for diagnosis of such cases. Aggressive systemic and local treatment can prolong survival, especially in patients with good performance status.

## 1. Introduction

Metastatic involvement of pancreas is rare and accounts for 2–5% of all pancreatic tumors [1]. Its incidence varies from 3% to 12% in autopsy series [2]. Tumors mostly metastatic to pancreas include renal cell carcinoma, melanoma, lung, colon, and gastric cancers. It usually appears as a late manifestation of disease and represents the diffuse spread of primary tumor [2].

Small cell lung cancer (SCLC) is a subtype of lung cancer with aggressive course and poor prognosis. Although it mostly spreads to the lungs, brain, bones, lymph nodes, and adrenal glands, it can involve almost all organs and tissues of the body. Although metastatic pancreatic involvement is a common finding of autopsy series in SCLC, metastasis-induced acute pancreatitis (MIAP) is very rare [2–4]. Here we reported a 50-year-old woman with SCLC who was admitted for attacks of acute pancreatitis and was diagnosed with MIAP.

## 2. Case Report

**Figure 1:**
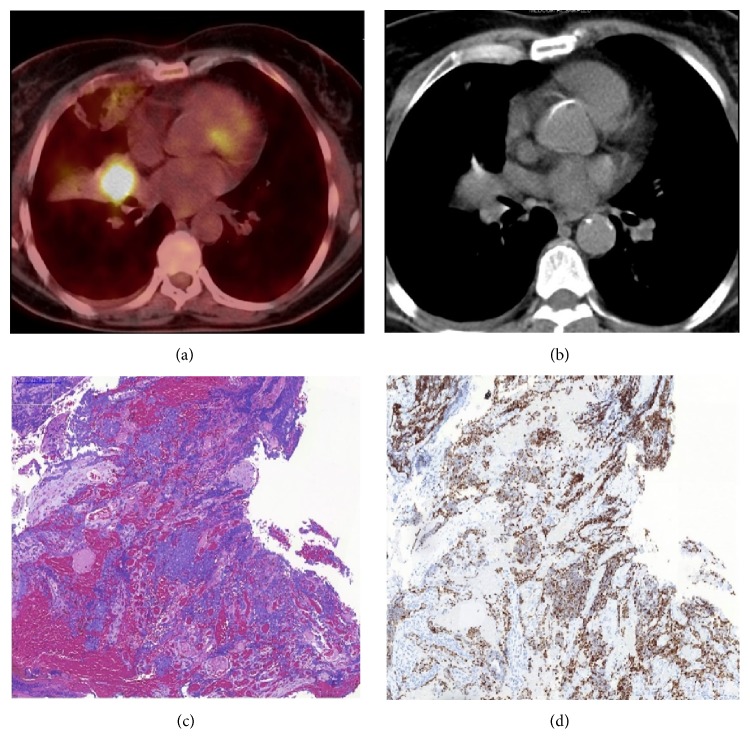
((a) and (b)) A hypermetabolic primary mass distal to the bronchus of the right middle lobe on PET-CT. (c) Infiltration of small cell carcinoma in transbronchial biopsy of the mass (H&E, ×10). (d) Thyroid transcription factor-1 (TTF-1) positive staining of tumor cells (TTF-1, ×10).

**Figure 2:**
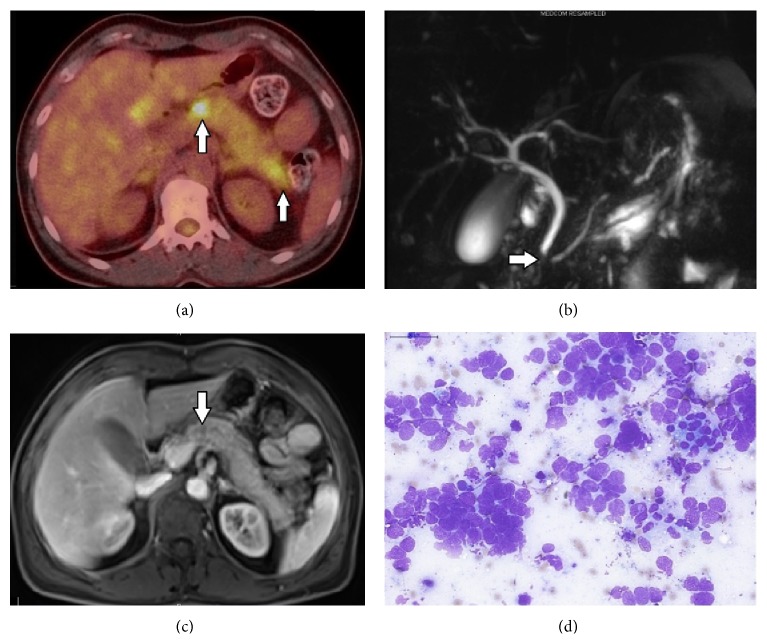
(a) Focal involvement areas of neck and tail of the pancreas on PET-CT (indicated by arrows). (b) Indistinct and nonuniformly circumscribed area of the pancreatic neck on abdominal MRI (arrow). (c) Segmental obliteration of the pancreatic duct on MRCP (arrow) and dilatation to its distal part. (d) Small cell carcinoma as shown by cytopathological examination of EUS-FNA taken from suspected area of the pancreas (MGG, ×40).

The medical work-up of a 50-year-old female patient who applied for chronic cough revealed a mass in the right lung. She had a 40-year pack smoking history and no history of alcohol abuse. Bronchoscopy showed an occlusive mass in the lateral segment bronchus of the right middle lobe and ^18^F-fluorodeoxyglucose (FDG) positron emission tomography-computed tomography (PET-CT) demonstrated a primary mass distal to the bronchus of the right middle lobe and hypermetabolic enlarged lymph nodes in the right lower and upper paratracheal region and the right supraclavicular region (Figures 1(a) and 1(b)). Bronchoscopic biopsy from the mass confirmed small cell carcinoma (Figures 1(c) and 1(d)). Patient's cranial magnetic resonance imaging (MRI) showed no metastasis, and then she was diagnosed with limited-stage SCLC and started cisplatin-etoposide concurrently with radiotherapy. Treatment was completed without major side effects and a PET-CT was performed after a month, which showed a full metabolic response to the chemoradiotherapy; during follow-up she was provided with prophylactic cranial radiation. The patient was admitted four months after completion of treatment for abdominal pain. The patient reported that she was hospitalized for diagnosis of acute pancreatitis for five days at an outside center two weeks ago; her complaints and amylase level which was initially high were regressed and improved after supportive therapy; however her abdominal pain progressively increased in the last two days. In the physical examination, she had localized pain in the epigastric and periumbilical area; the patient expressed that she felt the pain mostly on the back and lower back. Eastern Cooperative Oncology Group Performance Status (ECOG-PS) was 1 and there was no clinical finding of acute abdomen. The laboratory tests showed a mild leukocytosis and hyperamylasemia (780 U/L) with moderately high C-reactive protein. The patient's history involved no alcohol intake and cholelithiasis, and abdominal computed tomography (CT) demonstrated three metastatic lesions of 0.5–1 cm in diameter in the liver, nodular metastatic thickening in the right adrenal, and diffuse enlargement of the pancreas, and pancreatic ductus became slightly apparent. In addition to metastatic lesions described on abdominal CT, PET-CT showed abnormal focal FDG uptake in the neck and tail of pancreas with diffusely increased FDG uptake (Figure 2(a)). Magnetic resonance cholangiopancreatography (MRCP) revealed a segmental obliteration in the pancreatic duct and dilatation of its distal part (Figure 2(b)); postcontrast MRI sections demonstrated a poorly marginated hypointense area of around 1 cm at obliteration level in the pancreatic duct on the head-corpus junction of the pancreas (Figure 2(c)). Endoscopic ultrasonography (EUS) indicated a very indistinct area with irregular margins in the neck of pancreas and pancreatic duct interruption at this level. The cytopathological examination of EUS-guided fine-needle aspiration (EUS-FNA) from the lesion showed small cell carcinoma cells (Figure 2(d)). The patient was discussed at the tumor board, and a second-line chemotherapy with cisplatin and irinotecan (cisplatin 60 mg/m^2^ on day 1, irinotecan 60 mg/m^2^ on days 1, 8, and 15, every 4 weeks) and intensity-modulated radiotherapy (total dose of 30 Gy administered in daily 3-Gy fractions during 10 days) to pancreatic lesion were started concurrently. The patient's abdominal pain was relieved at the end of the first week of systemic chemotherapy and radiotherapy, and it completely disappeared after 3 weeks. The radiological studies performed after completion of second cycle of chemotherapy showed that metastatic lesions were regressed, and involvement of pancreas and dilatation of pancreatic duct disappeared. No pancreatic attacks were observed during follow-up. The patient is still alive at 14 months after her first diagnosis and 8 months after the first pancreatitis attack.

## 3. Discussion

The most common subtype of metastatic lung cancer to the pancreas is SCLC, followed by large cell carcinoma, squamous cell carcinoma, and adenocarcinoma [5, 6]. The incidence of metastasis to pancreas was reported to be 24–40% in patients with SCLC as revealed by postmortem studies [3, 4]. More than half of the patients are clinically asymptomatic and detected on radiological studies during follow-up. The most common symptoms are abdominal pain due to pancreatic invasion and jaundice associated with involvement of biliary tract [7]. In 1973, Levine and Danovitch were first to define acute pancreatitis associated with progression of the disease in a patient with SCLC [8]. Two series of 40 and 60 patients with SCLC reported the incidence of MIAP to be 7.5% and 3.3%, respectively [3, 9]. In a study by Stewart et al. that included the highest number of patients, only one patient was diagnosed with MIAP among 802 patients with lung cancer [10]. Although it is usually a late manifestation and present with extensive metastatic disease, it may rarely manifest as the initial symptom of the disease or as an isolated metastatic involvement [11].

The mechanism that is mostly held responsible for development of MIAP is obstruction/rupture of pancreatic duct due to compression of metastatic mass or enlarged regional lymph nodes. This is followed by vascular compromise/rupture secondary to tumor invasion. Other possible causes associated with development of acute pancreatitis in patients with SCLC include alcohol intake, cholelithiasis, hypercalcemia, and the use of chemotherapy agents such as cisplatin, ifosfamide, and vinorelbine. In cases where no etiological factor exists, paraneoplastic acute pancreatitis may be developed [6, 11].

Most of the cases of MIAP with SCLC in the literature were diagnosed clinically. Tissue diagnosis can be difficult in MIAP due to poor performance status of the patients and false negative rate of biopsy [12]. Therefore, there are no prospective studies on MIAP and most data comes from case reports and retrospective case series including patients with pancreatic metastasis.

The pancreatic metastasis associated with SCLC is commonly localized in the head and corpus of the pancreas. Radiologically, the most common types of pancreatic involvement are solitary metastatic mass (50–73%), diffuse pancreatic enlargement (15–44%), and multiple pancreatic nodules (5–10%), respectively [12, 13]. Ultrasonography's diagnostic value is low as the initial radiologic assessment and it can usually show accompanied biliary calculus and rarely dilatation of pancreatic duct. On a contrast-enhanced CT, a peripheral rim enhancement is typical for pancreatic metastasis and due to hypervascular pattern of tumor with respect to pancreatic parenchyma [6, 12, 13]. This pattern does not only provide detection of the localization of metastatic lesion but also allow differentiating primary pancreas adenocarcinoma that is seen as a mass with no uniformly contrast uptake [12, 13]. On MRI, pancreatic metastases appeared as hypointense and well-circumscribed lesions with peripheral involvement after contrast agent injection similar to CT. However, it was reported that uniform vascular pattern could be observed in lesions smaller than 1.5 cm on CT and MRI, and small lesions could not be demonstrated by imaging methods [12–15]. In our patient, abdominal CT, the initial imaging, showed no mass image in the pancreas except for radiologic findings of acute pancreatitis. However, PET-CT demonstrated two focal hypermetabolic areas with diffuse pancreatic involvement consistent with acute pancreatitis. There is limited data on the use of ^18^F-FDG PET for pancreatic metastases which is a very useful method for assessment of primary pancreatic tumors. There is only one relevant study by Sato et al. that included 573 patients with lung cancer who underwent PET-CT during initial staging or follow-up, and 11 patients were then diagnosed with pancreatic metastasis [16]. In 3 of these 11 patients, no visible radiologic lesions were observed in the pancreas with standard transaxial CT.

EUS is probably the best method for the evaluation of pancreatic masses. The appearance of primary pancreatic tumors is similar to pancreatic metastases on EUS; however metastatic lesions were reported to usually have more well-defined borders than primary tumors [17]. In our case, MIAP was caused by obstruction of pancreatic duct associated with invasion of a small metastasis localized in the pancreatic neck. In contrast to the literature, the focal involvement area in the pancreatic neck on PET-CT appeared indistinct and nonuniformly circumscribed on both MRI and EUS. Various studies reported diagnostic accuracy of EUS-FNA for pancreatic metastases to be 89–92% [17–19]. EUS-FNA appears to be the most effective method for tissue diagnosis of small pancreatic masses in particular [19]. In our case, the pancreatic lesion was cytopathologically confirmed to be metastasis of SCLC by EUS-FNA.

There is no standard treatment approach for MIAP in patients with SCLC. Endoscopic intrapancreatic stent implantation is a palliative solution, but in some cases it can improve performance status of the patients and consequently make them suitable for receiving chemotherapy [6, 20]. Radiotherapy can be used for palliation of symptoms. Because SCLC is a chemosensitive tumor, a systemic chemotherapy improves outcomes. The survival time varies from 4 months to 6 months after developing MIAP in patients receiving chemotherapy, and the survival of patients who receive no treatment is around 2–4 weeks [9, 10, 21]. A study by Lin et al. found major predictors of survival to be duration of high amylase levels, performance status when MIAP occurred, and the length of chemotherapy after diagnosis of MIAP [22]. The study by Liu et al. reported a better overall survival in patients with a good performance status and receiving systemic chemotherapy. In our case, the longer survival was probably due to a good performance status at diagnosis of MIAP and good tumor response achieved by early initiation of aggressive treatment. The main symptom of the patient was abdominal pain due to pancreatic involvement; however there was also systemic disease with adrenal and liver metastases. Consequently, radiotherapy for the palliation of pain and systemic chemotherapy were started concurrently. The patient experienced no major treatment-related side effect and achieved complete pain relief and systemic disease control.

As a result, MIAP is a rare clinical entity that may occur in the course of disease or as an initial symptom in patients with SCLC. It should be noted that standard imaging methods may not demonstrate a mass image in some cases. In such cases, PET-CT and EUS-FNA can be used to confirm the diagnosis. Rapid and early diagnosis, systemic chemotherapy, and local treatments may have an influence on the palliation of symptoms and survival. An aggressive treatment may prolong survival particularly in patients with good performance status.
